# EPIDEMIOLOGICAL STUDY ON LISFRANC INJURIES

**DOI:** 10.1590/1413-785220172501168995

**Published:** 2017

**Authors:** MARCEL FARACO SOBRADO, GUILHERME HONDA SAITO, MARCOS HIDEYO SAKAKI, PEDRO AUGUSTO PONTIN, ALEXANDRE LEME GODOY DOS SANTOS, TÚLIO DINIZ FERNANDES

**Affiliations:** 1. Universidade de São Paulo, Faculdade de Medicina, Hospital das Clinicas, Instituto de Ortopedia e Traumatologia, São Paulo, SP, Brazil.

**Keywords:** Tarsal joints/injury, Metatarsal bones/injuries, Foot injuries/surgery, Dislocations/surgery

## Abstract

**Objective:**

To analyze the characteristics of patients with Lisfranc injuries and their associated fractures***.***

**Methods:**

This is a retrospective analysis on 42 patients with Lisfranc injuries hospitalized at Instituto de Ortopedia e Traumatologia do Hospital das Clínicas da Faculdade de Medicina da Universidade de São Paulo, between 2006 and 2010. Parameters on patient profile, risk factors, fracture characteristics, data on treatment and acute complications were analyzed***.***

**Results:**

Analysis of 42 cases showed that in our sample, men were more affected than women, with a ratio of 4.25:1. The most frequent trauma mechanism was car accident, followed by motorcycle accident. The most frequent type of injury was isolated lesion type B of Quenu and Kuss classification, representing 50% of cases. The most common fracture on the sample was the second metatarsal bone, with 16 cases, followed by cuboid bone fracture. Among the 42 cases, 17% had exposed fractures and 33 patients presented other associated fractures. The mean time elapsed between the trauma and definitive treatment was 6.7 days, while the mean length of hospital stay was 13.8 days. Six patients presented acute postoperative complications***.***

**Conclusion:**

Lisfranc injuries are more common in men undergoing automobile trauma. The prevalence of associated fractures is a frequent finding and the hospital stay may be longstanding.***Level of Evidence IV, Case Series.***

## RESUMO

**Objetivo:**

*Analisar o perfil de pacientes com lesões de Lisfranc, as características das lesões e fraturas associadas.*

**Métodos:**

*Trata-se de uma análise retrospectiva com 42 pacientes com lesões de Lisfranc internados no Instituto de Ortopedia e Traumatologia do Hospital das Clínicas da Faculdade de Medicina da Universidade de São Paulo entre 2006 e 2010. O perfil dos pacientes, características das lesões, fraturas associadas, dados sobre o tratamento e complicações agudas foram analisados.*

**Resultados:**

*Nesta amostra, os homens foram mais afetados do que as mulheres, com uma proporção de 4,25:1. O mecanismo de trauma mais frequente foi acidente de carro, seguido por acidente com moto. O tipo de lesão mais frequente foi a lesão isolada tipo B de Quenu e Kuss, representando 50% dos casos. A fratura mais comumente encontrada foi a do segundo metatarso, com 16 casos, seguido pela fratura do osso cubóide. Entre os 42 casos estudados, sete foram fraturas expostas e 33 pacientes apresentaram fraturas associadas. O tempo médio entre o trauma e o tratamento definitivo foi de 6,7 dias. O tempo médio de permanência hospitalar foi de 13,8. Seis pacientes apresentaram complicações pós-operatórias agudas.*

**Conclusão:**

***As lesões de Lisfranc são mais comuns em homens submetidos a trauma automobilístico. A prevalência de fraturas associadas é um achado frequente e o tempo de permanência hospitalar pode ser prolongado. Nível de Evidência IV, Série de Casos:***

## INTRODUCTION

The term Lisfranc injury is used to refer to injuries involving damage to the tarsometatarsal joint. The term covers a broad spectrum of injuries ranging from damage to the ligaments alone to fractures and fracture-dislocations. Retrospective studies have shown that up to one-third of these injuries go unnoticed during initial assessment.^1^ ^-^ ^5^

According to the literature, Lisfranc injuries are more common around the third decade of life, and are 2-4 times more common in men. Nevertheless, Lisfranc injuries are relatively uncommon, representing approximately 0.2% of all fractures, and are generally associated with fractures of the tarsal and metatarsal bones.^6^

Although fracture of the cuneiform bones is common, the most common fracture in the tarsometatarsal complex occurs at the base of the second metatarsal. Fractures of the navicular, cuboid, and other metatarsals are less common.^7^ ^-^ ^10^

The literature indicates that the vast majority (87.5%) of these injuries are closed, and that up to one-third of these injuries occur in athletes during low-energy sports trauma.^11^ ^,^ ^12^

Two trauma mechanisms are described: high and low energy. High-energy injuries may result from direct or indirect trauma. Application of a direct load to the dorsal surface of the joint complex, which occurs from crushing or the impact of an object on the static foot, may result in injuries to the bones or to the ligaments through the joint line. The pattern may vary depending on where the force is applied. This type of injury can cause significant damage to the soft tissues, compromising the treatment sequence.^13^ ^-^ ^15^ However, the most common mechanism is indirect injury, which is characterized by a longitudinal force on one foot in plantar flexion. Bone injuries and more severe instabilities usually result from high-energy trauma such as falls from heights or car crashes. The injuries may be evident, but in a considerable portion of patients, spontaneous reduction may occur after the trauma, thus masking the underlying instability. Low-energy injuries include sports traumas, for example in American football.^2^ ^,^ ^14^ ^,^ ^16^ ^-^ ^18^ The diagnosis is made by evaluating anteroposterior, lateral, and oblique X-rays of the foot bearing weight. It is important to stress that weight bearing X-rays be done, because in some cases, as mentioned previously, the instability will only be evident after load is placed on the feet.^14^ ^,^ ^19^ The most common finding is diastasis between the base of the first and second metatarsals. Any fracture of the first three metatarsals increases suspicion of the existence of a Lisfranc injury. Computed tomography plays an important role in diagnosis by detecting small fractures and deviations and identifying possible associated injuries.^7^ ^,^ ^20^

One of the classifications used most commonly in assessing Lisfranc injuries is that of Quenu and Kuss,^21^ which divides Lisfranc injuries into types A, B, and C. Type A involves homolateral rupture, in which all metatarsals move in the same direction. In type B injuries, there is an isolated rupture which can involve the first metatarsal or the smaller rays. Type C is divergent displacement, where the first ray and lesser rays are dislocated in opposite directions.

To define the treatment for a Lisfranc injury, assessment of joint stability is essential. Unstable injuries require surgical treatment with anatomical reduction and stable fixation.

It should be emphasized that many patients with injuries restricted to the ligaments develop chronic pain and instability, although anatomical reduction and stable fixation are achieved.^22^ ^,^ ^23^

The most frequent acute complications are acute compartmental syndrome, vascular damage, skin necrosis, and superficial infections.^24^

The objective of this study was to investigate the epidemiology of Lisfranc injuries found in hospitalized patients.

## MATERIALS AND METHODS

The study was approved by the Institutional Review Board under process number 924 and the record CAPpesq/HC 9335.

All medical records for patients hospitalized with foot and ankle fractures between January 2006 and December 2010 were analyzed. Review of these records identified 42 cases of Lisfranc injuries. The parameters analyzed were age, gender, laterality, exposure, injury mechanism, fracture type, classification, associated injuries, emergency treatment, definitive treatment, time between trauma and definitive treatment, length of hospital stay, and acute post-operative complications.

## RESULTS

**Figure 1:**
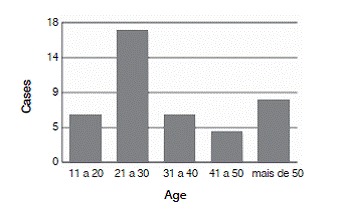
Age range.

Among the 42 patients studied, we observed that these injuries occurred predominantly in men, who accounted for 81% of the patients. The mean age of the patients studied was 35.5 years, ranging from 19 to 66 years. (Figure 1) The left side was more frequently affected, corresponding to 24 patients, or 57% of the cases. 

**Figure 2:**
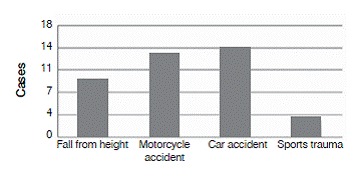
Injury mechanism.

The most common injury mechanism was automobile accidents in 35.8% of cases, followed by motorcycle accidents (33.3%), falls from height (23.0%), and sports accidents (7.7%). (Figure 2) Of the total, seven (16.7%) patients experienced multiple traumas.

Of the 42 cases, 35 presented closed injuries and 7 had open injuries (16.7%). 

According to the classification of Quenu and Kuss, 43% of the patients presented homolateral injuries (type A), 50% had isolated injuries (type B), and 7% had divergent injuries (type C).

**Figure 3:**
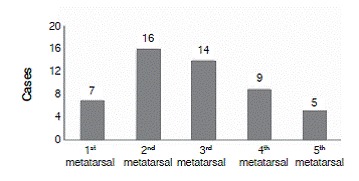
Fractured metatarsal.

**Figure 4:**
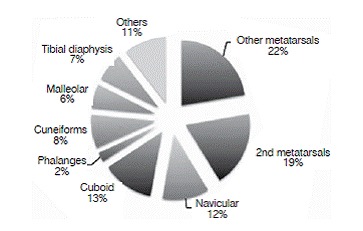
Associated fractures.

Seventy-eight percent of the patients had fractures associated with an injury to the Lisfranc complex. The most prevalent associated fracture was of the second metatarsal, which was present in 16 of the 42 individuals studied (38%). The third metatarsal was involved in 14 cases (33%), followed by the fourth metatarsal in 9 cases (21%). Other associated fractures were of the cuboid bone in 11 cases (26%), the navicular bone in 10 cases (24%), the cuneiforms in seven cases (17%), the tibia diaphysis in six cases (14%), and malleolar fractures in five cases (12%). (Figures 3 and 4).

Of the 42 patients studied, in five cases conservative treatment was chosen. Of the 37 cases treated surgically, primary arthrodesis was performed in two patients, and reduction and internal fixation were performed in the remaining 35. In relation to fixation method, in 21 cases cannulated screws alone or associated with Kirschner wires were used, eight cases were treated with only Kirschner wires, and plates were used in six cases. Two cases required an external mini-fixator to maintain length.

The average length of time between the temporary treatment and definitive fixation was 6.7 days, varying from 0 to 28 days.

In terms of acute complications, we observed 6 cases of post-operative infection (14%). Of these, one case required a microsurgical flap to cover the wound. One case of superficial skin dehiscence occurred, one case of deep venous thrombosis.

**Figure 5:**
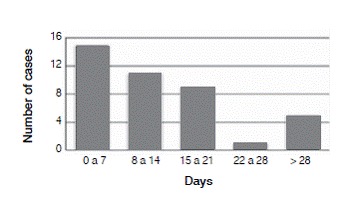
Duration of hospitalization.

The median hospital stay was 13.8 days, ranging from 0 to 55 days. (Figure 5)

## DISCUSSION

This present study was conducted over a period of four years (2006 to 2010) in a tertiary hospital specializing in orthopedics and traumatology which treats a significant number of emergency cases. It is consequently noted that Lisfranc injury has a low prevalence in the universe of surgically-treated fractures, with a mean frequency of 10.5 cases per year in this service. 

We note that, in our study, this type of injury predominantly affected men (81%) and average patient age was 35.5 years. This is a young and economically active population which is affected by significant financial and social losses from this type of injury. In epidemiological terms, it is compatible with the literature, which reports greater incidence of this injury around 30 years of age and 75% predominance in men.^7^ ^,^ ^25^

Motor vehicle accidents accounted for 69% of the total trauma mechanisms, with auto accidents slightly more common than motorcycle accidents. However, sports accidents corresponded to only 7.7%, a lower prevalence than suggested by the literature. Benirschke et al.^12^ showed in their study that this type of mechanism could account for up to 30% of cases. This discrepancy may be attributed to the different epidemiological profile of the patients treated at our institution. Since the Hospital das Clínicas is a reference hospital, there is a predominance of high-energy trauma such as auto and motorcycle accidents.

The left side was more affected (57%) and open fractures were present in 17% of cases. This rate is in accordance with the international literature. According to Stavlas and Miswan, the prevalence of open fractures in this type of injury may vary from 12.5 to 26%.^11^ ^,^ ^25^

In 33 cases (78.6%), associated fractures were present, similar to the findings by Aitken et al.^26^ and Meyerson et al.,^2^ who also observed a higher frequency of fractures at the base of the second metatarsal and the cuboid bone.

In only five cases (12%) was conservative treatment chosen, which demonstrates the predominance of surgical treatment of this type of injury. Definitive surgical treatment occurred an average of six days after the initial trauma. Definitive treatment for Lisfranc injuries can be postponed in situations with large soft tissue injuries, acute compartment syndrome, and open fractures secondary to crushing. Consequently, there is no consensus in the literature with regard to the ideal period for conducting the definitive procedure.^24^

As for acute complications associated with fracture or treatment, the post-operative infection rate in our study was 14.3%, higher than that reported in the literature, which ranges from 4.8% to 7.3%.^27^ ^,^ ^28^ One case of post-operative infection progressed and required a microsurgical flap for coverage. Only one patient developed deep venous thrombosis.

The mean hospital stay was 13.8 days. This lengthy average hospitalization can be primarily attributed to the patients with multiple traumas whose definitive treatments were delayed, as well as to acute post-operative complications such as infections and complications of the surgical wound. This prolonged period of hospitalization generates high costs for the public health system and also lowers bed turnover.

## CONCLUSION

Although rare, Lisfranc injuries found in patients hospitalized in a high-complexity service occurred in young men who were involved in motor vehicle accidents, and most cases involved an associated fracture of another bone in the foot. The incidence of acute complications is high and hospital stays are lengthy.

## References

[1] Rammelt S, Schneiders W, Schikore H, Holch M, Heineck J, Zwipp H (2008). Primary open reduction and fixation compared with delayed corrective arthrodesis in the treatment of tarsometatarsal (Lisfranc) fracture dislocation. J Bone Joint Surg Br.

[2] Myerson MS, Fisher RT, Burgess AR, Kenzora JE (1986). Fracture dislocations of the tarsometatarsal joints: end results correlated with pathology and treatment. Foot Ankle.

[3] Perron AD, Brady WJ, Keats TE (2001). Orthopedic pitfalls in the ED: Lisfranc fracturedislocation. Am J Emerg Med.

[4] Philbin T, Rosenberg G, Sferra JJ (2003). Complications of missed or untreated Lisfranc injuries. Foot Ankle Clin.

[5] Goossens M, De Stoop N (1983). Lisfranc's fracture-dislocations: etiology, radiology, and results of treatment: a review of 20 cases. Clin Orthop Relat Res.

[6] Wright MP, Michelson JD (2013). Lisfranc injuries. BMJ.

[7] Desmond EA, Chou LB (2006). Current concepts review: Lisfranc injuries. Foot Ankle Int.

[8] Nunley JA, Vertullo CJ (2002). Classification, investigation, and management of midfoot sprains: Lisfranc injuries in the athlete. Am J Sports Med.

[9] Lattermann C, Goldstein JL, Wukich DK, Lee S, Bach BR (2007). Practical management of Lisfranc injuries in athletes. Clin J Sport Med.

[10] Coetzee C (2008). Making sense of Lisfranc injuries. Foot Ankle Clin.

[11] Stavlas P, Roberts CS, Xypnitos FN, Giannoudis PV (2010). The role of reduction and internal fixation of Lisfranc fracture-dislocations: a systematic review of the literature. Int Orthop.

[12] Benirschke SK, Meinberg E, Anderson SA, Jones CB, Cole PA (2012). Fractures and dislocations of the midfoot: Lisfranc and Chopart injuries. J Bone Joint Surg Am.

[13] Trevino S, Kodros S (1995). Controversies in tarsometatarsal injuries. Orthop Clin N Am.

[14] Vuori J, Aro H (1993). Lisfranc joint injuries: trauma mechanisms and associated injuries. J Trauma.

[15] Wilson DW (1972). Injuries of the tarso-metatarsal joints. J Bone Jone Surg Br.

[16] Curtis M, Myerson M, Szura B (1993). Tarsometatarsal joint injuries in the athlete. Am J Sports Med.

[17] Faciszewski T, Burks R, Manaster B (1990). Subtle injuries of the Lisfranc joint. J Bone Jone Surg Am.

[18] Meyer S, Callaghan J, Albright J, Crowley ET, Powell JW (1994). Midfoot sprains in collegiate football players. Am J Sports Med.

[19] Kalia V, Fishman EK, Carrino JA, Fayad LM (2012). Epidemiology, imaging, and treatment of Lisfranc fracture-dislocations revisited. Skeletal Radiol.

[20] Hawkes NC, Flemming DJ, Ho VB (2007). Subtle Lisfranc injury: low energy midfoot sprain. Mil Med.

[21] Quenu E, Kuss G (1909). Etude sur les luxutations du metatarse (luxations metatarso-tarsiennes) du diastasis entre le 1. et le 2. metatarsien. Rev Chir Paris.

[22] Kuo R, Tejwani N, DiGiovanni C, Holt SK, Benirschke SK, Hansen ST (2000). Outcome after open reduction and internal fixation of Lisfranc joint injuries. J Bone Jone Surg Am.

[23] Mulier T, Reynders P, Sioen W, van den Bergh J, de Reymaeker G, Reynaert P (1997). The treatment of Lisfranc injuries. Acta Orthop Belg.

[24] Bellabarba C, Barei DP, Sanders RW, Coughlin MJ, Mann RA, Saltzman CL (2007). Dislocations of the foot.

[25] Miswan MF, Singh VA, Yasin NF (2011). Outcome of surgically treated Lisfranc injury: a review of 34 cases. Ulus Travma Acil Cerrahi Derg.

[26] Aitken AP, Poulson D (1963). Dislocations of the tarsometatarsal joint. J Bone Joint Surg Am.

[27] Zhu H, Zhao HM, Yuan F, Yu GR (2011). Effective analysis of open reduction and internal fixation for the treatment of acute Lisfranc joint injury. Zhongguo Gu Shang.

[28] Groulier P, Pinaud JC (1970). Tarso-metatarsal dislocations (10 cases). Rev Chir Orthop Reparatrice Appar Mot.

